# Where are race-based oral health inequities bound? Protocol for a systematic review on interventions to tackle racial injustice in dental outcomes

**DOI:** 10.1186/s13643-022-01911-w

**Published:** 2022-03-07

**Authors:** João L. Bastos, Helena M. Constante, Helena S. Schuch, Dandara G. Haag, Sonia Nath, Roger K. Celeste, Carol C. Guanizo-Herreño, Mary J. McCallum, Lisa M. Jamieson

**Affiliations:** 1 Federal University of Santa Catarina, Florianópolis, Brazil; 2 Federal University of Pelotas, Pelotas, Brazil; 3 Australian Research Centre for Population Oral Health, The University of Adelaide, Adelaide, Australia; 4 Department of Preventive and Social Dentistry, Federal University of Rio Grande do Sul, Porto Alegre, Brazil; 5 Facultad de Odontología, Universidad Nacional de Colombia, Bogotá, Colombia; 6 Dentist of Cree Descent, Winnipeg, Canada

**Keywords:** Racial injustice, Health inequities, Interventions, Oral health, Systematic literature review

## Abstract

**Background:**

Only three literature reviews have assessed the impact of interventions on the reduction of racial inequities in general health to date; none has drawn from attempts at promoting racial oral health equity. This protocol aims to increase transparency and reduce the potential for bias of an ongoing systematic review conceived to answer the following questions: Are there any interventions to mitigate racial oral health inequities or improve the oral health of racially marginalized groups? If so, how successful have they been at promoting racial oral health equity? How do conclusions of previous reviews change by taking the findings of oral health interventions into account?

**Methods:**

Reviewed studies must deploy interventions to reduce racial gaps or promote the oral health of groups oppressed along ancestral and/or cultural lines. We will analyze randomized clinical trials, natural experiments, pre-post studies, and observational investigations that emulate controlled experiments by assessing interactions between race and potentially health-enhancing interventions. Either clinically assessed or self-reported oral health outcomes will be considered by searching for original studies in MEDLINE, LILACS, PsycInfo, SciELO, Web of Science, Scopus, and Embase from their earliest records to March 2022. Upon examining abstracts of conference proceedings, trial registries, reports of related stakeholder organizations, as well as contacting researchers for unpublished data, we will identify studies in the grey literature. If possible, we will carry out a meta-analysis with subgroup and sensitivity analysis, including formal meta-regression, to address potential heterogeneity and inconsistency among selected studies.

**Discussion:**

Conducting a systematic review of interventions to mitigate racial oral health inequities is crucial for determining which initiatives work best and under which conditions they succeed. Such knowledge will help consolidate an evidence base that may be used to inform policy and practice against persistent and pervasive racial inequities in general and oral health.

**Systematic review registration:**

This protocol has been registered at the International Prospective Register of Systematic Reviews, under the identification number CRD42021261450.

## Background

Research on racial oral health inequities has moved apace in the past few decades. Since the 1990s, tallies of articles on race and poor oral health have increased exponentially, outnumbering studies focused on, for instance, class-based oral health inequities [1]. This quantitative growth has not been followed by the construction of a compelling anti-racist narrative, however [2]. Amid technical developments in measuring racial [3, 4] and socioeconomic [5, 6] gaps in dental outcomes, oral health scholars have mostly relied on biological conceptions of race, which often ignore the major root cause of race-based oral health inequities, i.e., racism [2]. Indeed, as a macro-level system that differentially allocates power and social resources along racial lines, racism is almost entirely absent from the oral health literature [1, 2, 7]. Unsurprisingly, this general trend echoes the main debates taking place in scholarly medical journals, which draw heavily from race as a context-free, fixed, individual-level characteristic [8–10].

The persistent and ethically charged character of all forms of racial inequity [11] has prompted researchers, policymakers, and other key social actors to devise interventions aimed at mitigating race-based health inequities. In the field of dentistry, for example, studies have shown that racial oral health inequities may be attenuated by a range of different measures. While some of them center around individual oral health-related behaviors and the family context, others address broader processes and institutions, such as the organizational structure of health care systems. One recent publication [12] suggested that racial inequities in health care accessibility and perceived quality of care were lower among specialized dental clinics with a health-service ombudsman, compared to oral health care centers where such an official had not been appointed. A multipronged intervention—i.e., provision of dental treatment to mothers, application of fluoride varnish to children, and use of motivational interviewing, together with anticipatory guidance—proved to be effective in reducing dental caries levels among Indigenous Australian children [13]. Children who received the intervention earlier had fewer dental caries than those who did so at a later point in time, the trajectory of dental caries was lower than that predicted by simulation analyses of no intervention, and the beneficial oral health effects were more pronounced for children receiving the intervention in the earlier rather than later infancy.

These and other initiatives notwithstanding, only three literature reviews [8, 14, 15] have discussed their findings to date. The earliest one was published in 2005 [14]. As well as providing an overview of theoretical issues that apply to the empirical study and public policy of anti-racism, Paradies [14] discussed a handful of social psychological approaches for combating racism and its disproportionate toll on Aboriginal Australians. Williams and Mohammed’s work [15] emphasized, on the other hand, African Americans living in the USA, as well as a host of successful interventions against racism at its multiple levels (e.g., institutional, cultural, interpersonal) and the related health inequities. By critically reviewing the literature on racism and general health, Bailey et al. [8] advocated for place-based, multisector, equity-oriented initiatives, which would effectively counteract structural racism and eventually advance health equity. Assessing the health impacts of large-scale interventions, such as the Purpose-Built Communities [16] and the Moving to Opportunity Study [17], encouraging policy reform, and training the next generation of health professionals would be key to eradicating racial health inequities, these authors argued.

None of the reviews mentioned above has, however, drawn from interventions devised to promote racial equity in oral health or improve unfavorable dental outcomes among specific racially marginalized groups. Though general and oral health share common determinants and may be enhanced by similar interventions [18], the fact that oral health initiatives have not been included in these previous works is concerning for at least three main reasons. First and foremost, given that oral health services are usually separated from mainstream healthcare systems, racialized minorities are faced with even more restricted access to dental than to medical care [19, 20]. Second, the limited availability of dental care is often coupled with professional practices that are deleterious for racially marginalized groups, including little to no cultural competency and unwelcoming behaviors that do not accommodate minorities’ specific needs, such as limiting the number of the patient’s supporting personnel [21]. Third, extant evidence indicates that the comparatively higher inequity in access to quality dental care extends to adverse dental outcomes. Earlier studies [22] have shown that socioeconomic inequities in oral health tend to be greater than inequities in general health, particularly when comparing decayed and missing teeth with obesity and hypertension. Thus, it remains to be known whether conclusions of the previous literature reviews [8, 14, 15] hold when initiatives aimed at reducing racial oral health inequities are taken into account.

This protocol aims to increase transparency and reduce the potential for bias [23] of an ongoing systematic review of original studies aiming to mitigate race-based oral health inequities. The main objectives of the review study are to (1) identify initiatives to reduce racial gaps in oral health or improve the dental status of specific racially marginalized groups, and (2) determine the effectiveness of these interventions in promoting racial oral health equity. The discussion around the paucity of oral health interventions—which justifies their omission from the three reviews mentioned above [8, 14, 15]—will be informed by the ecosocial theory of disease distribution, as the scarcity of knowledge on the topic is neither neutral nor harmless [24]. Ignorance of the overall effects of these much-needed dental interventions is, in fact, one way to maintain the *status quo* of racial and other forms of oral health inequity [10].

## Methods

### Protocol and registration

This protocol was registered at the International Prospective Register of Systematic Reviews (https://www.crd.york.ac.uk/prospero/), under this number of identification: CRD42021261450. While the present paper was prepared following the “Preferred Reporting Items for Systematic Review and Meta-analysis Protocols” [25], the ensuing systematic review and meta-analysis will be prepared according to the guidelines of the “Preferred Reporting Items for Systematic Reviews and Meta-Analysis” [26].

### Review questions

The systematic review that will emerge from this protocol has been conceived to answer the following three questions: Are there any interventions to mitigate racial oral health inequities or improve the oral health of racially marginalized groups? If so, how successful have they been at promoting racial oral health equity? How do conclusions of previous reviews change by taking the findings of oral health interventions into account?

### Eligibility criteria

#### Population

Our focus is population groups oppressed along ancestral and/or cultural lines, most notably those that have been defined on the basis of race, ethnicity, indigeneity, caste, religion, tribe, origin, nationality, immigration status, and/or language in the field of dentistry [1, 2, 7].

#### Intervention

We take racial oral health inequities as the unfair, avoidable, and excessive burden of any adverse oral health outcome that racialized minorities often bear, relative to the privileged groups with whom they relate [2, 27]. Based on this definition, we will analyze interventions that either foster racial equity or focus on mechanisms linking race-based oppression with racial oral health inequities. As such, interventions might combat either intrapersonal, interpersonal, or institutional/systemic/structural racism and may examine different units of analysis, from individuals to population aggregates. We will also include any type of intervention designed to improve the oral health of specific racially marginalized groups, as well as observational investigations that emulate controlled experiments by assessing interactions between race and potentially health-enhancing interventions. Animal or laboratory studies, qualitative studies, case reports, clinical conference studies, consensus development studies, editorials, letters/commentaries, and scientific integrity reviews will be excluded.

#### Comparator

The oral health status of racially marginalized groups is sometimes compared with that of racially-dominant groups, whose defining characteristics vary according to place, time, and sociocultural specificities. For example, while in contemporary India the most powerful groups emerge out of a complex caste system, in some former Western colonies, including Brazil, racially hegemonic groups are mostly composed of descendants of European settlers. However, since a number of original studies avoid these comparisons, and instead assess the success of interventions among only racially minoritized groups, we will also include this latter type of investigation in the review.

#### Outcome

We will take into consideration both clinically assessed and self-reported oral health outcomes, such as dental caries, periodontal diseases, tooth loss, toothache, poor self-rated oral health, and poor oral health-related quality of life. Interventions aimed at improving knowledge, attitudes, and practices related to oral health will not be assessed, neither those aiming to increase access to or use of dental services. According to Phelan and Link, decreased access to and use of (oral) health services may be taken as a mechanism linking racism with race-based (oral) health inequities [28].

### Information sources

Electronic searches of MEDLINE via PubMed, LILACS, PsycInfo, SciELO, Web of Science, Scopus, and Embase will be carried out. The searches will encompass publications from the earliest records of these bibliographic databases to March 2022, with search results updated prior to the final analysis. Upon examining abstracts of conference proceedings, trial registries, reports of related stakeholder organizations, as well as contacting researchers for unpublished data, we will identify studies in the gray literature. We will consider publications available in all idioms, with papers published in languages other than English, Spanish, and Portuguese translated into English with Google Translator in light of the language capacities of the research team. Authors will be contacted in case an intelligible translation into English cannot be obtained with the aforementioned web-based tool.

### Search query

A preliminary search strategy template for use in MEDLINE via PubMed is provided in Table 1 below. This template has no pre-specified limits and will be adapted for use in the other bibliographic databases mentioned above.

**Table 1:** Preliminary search query for use in the MEDLINE via PubMed

	Population	Intervention	Comparison group	Outcome	Studies to be excluded by design
Search terms	(“continental population groups”[mesh] OR “population groups”[mesh] OR “ethnic groups”[mesh] OR “arctic regions”[mesh] OR “minority groups”[mesh] OR “indigenous peoples”[mesh] OR “skin pigmentation”[mesh] OR “skin color”[tiab] OR “skin colo*”[tiab] OR “people of color”[tiab] OR “race*”[tiab] OR “racial group”[tiab] OR “ethnicit*”[tiab] OR “ethnic*”[tiab] OR “ethnic group*”[tiab] OR “ethnic minorit*”[tiab] OR “immigrant*”[tiab] OR “caste*”[tiab] OR “tribe*”[tiab] OR “tribal”[tiab] OR “origin”[tiab] OR “nationalit*”[tiab] OR “religious group*”[tiab] OR “black*”[tiab] OR “aborigin*”[tiab] OR “indigen*”[tiab] OR “first nation*”[tiab] OR “pacific islander*”[tiab] OR “torres strait islander*”[tiab] OR “alaska*”[tiab] OR “aleut*”[tiab] OR “amerind*”[tiab] OR “arctic”[tiab] OR “aymara”[tiab] OR “bushmen”[tiab] OR “chukchi”[tiab] OR “chukotka”[tiab] OR “circumpolar”[tiab] OR “eskimo*”[tiab] OR “greenland*”[tiab] OR “hmong”[tiab] OR “indian*”[tiab] OR “inuit*”[tiab] OR “inupiaq”[tiab] OR “inupiat”[tiab] OR “khanty”[tiab] OR “maori*”[tiab] OR “mapuche”[tiab] OR “metis”[tiab] OR “native*”[tiab] OR “navajo”[tiab] OR “nenets”[tiab] OR “quechua”[tiab] OR “sami”[tiab] OR “samoan”[tiab] OR “siberia*”[tiab] OR “skold”[tiab] OR “xingu*”[tiab] OR “yup’ik”[tiab] OR “zuni”[tiab])	(“anti-racism”[tiab] OR “race relations”[mesh] OR “race factors”[mesh] OR “racism”[mesh] OR “social discrimination”[mesh] OR “prejudice”[mesh] OR “racial bias”[tiab] OR “racial mistreatment”[tiab] OR “racial injustice”[tiab] OR “health equity”[mesh] OR “racial equity”[tiab] OR “racial inequit*”[tiab] OR “racial inequalit*”[tiab] OR “differential*”[tiab] OR “preventive dentistry”[mesh] OR “dental prophylaxis”[mesh] OR “pit and fissure sealants"[mesh] OR “cariostatic agents”[mesh] OR “fluoride*”[tiab] OR “fluoridation”[mesh] OR “dentifrices”[mesh] OR “toothpastes”[mesh] OR “health education”[mesh] OR “counseling”[mesh] OR “health behavior”[mesh] OR “health promotion”[tiab] OR “government programs”[mesh] OR “public policy”[mesh] OR “public assistance”[mesh] OR “health policy”[tiab] OR “intervention*”[tiab] OR “welfare”[tiab] OR “preventive health services”[mesh] OR “dental health services”[mesh] OR “delivery of health care”[mesh] OR “dental care”[mesh] OR “dental check up*”[tiab] OR “dental checkups”[tiab] OR “dental visit*”[tiab] OR “dental insurance”[tiab] OR “indigenous health service”[tiab] OR “motivational interview*”[tiab] OR “oral health literacy”[tiab] OR “impact”[tiab] OR “oral impacts”[tiab] OR “chewing difficulties”[tiab])	Does not apply	(“oral diseases”[tiab] OR “stomatognathic diseases”[mesh] OR “tooth disease”[tiab] OR “mouth disease”[tiab] OR “oral health”[mesh] OR “oral health”[tiab] OR “dental health”[tiab] OR “xerostomia”[mesh] OR “mastication”[mesh] OR “mouth neoplasms”[mesh] OR “leukoplakia, oral”[mesh] OR “oral cancer"[tiab] OR “oral pathology”[tiab] OR “oral dysplasia”[tiab] OR “exostosis”[tiab] OR “fluorosis, dental”[mesh] OR “dental fluorosis”[tiab] OR “enamel defect”[tiab] OR “dental enamel hypoplasia”[mesh] OR “periodontal diseases”[mesh] OR “periodontal attachment loss”[mesh] OR “periodontal pocket”[mesh] OR “gingivitis”[tiab] OR “oral hygiene”[mesh] OR “oral hygiene index”[mesh] OR “dental plaque index”[mesh] OR “tooth injuries”[mesh] OR “dental trauma”[tiab] OR “toothache”[mesh] OR “oral pain”[tiab] OR “facial pain”[mesh] OR “dmf index"[mesh] OR “dental caries”[mesh] OR “dental caries”[tiab] OR “tooth loss”[mesh] OR “tooth loss”[tiab] OR “dental loss”[tiab] OR “mouth, edentulous”[mesh] OR “edentulism”[tiab] OR “oidp”[tiab] OR “ohip*”[tiab] OR “gohai”[tiab] OR “ecohis”[tiab] OR “ohrql”[tiab] OR “temporomandibular joint disorders”[mesh] OR “esthetics, dental”[mesh] OR “malocclusion”[mesh] OR “index of orthodontic treatment need”[mesh] OR “iotn”[tiab])	(“models, animal”[mesh] OR “observational study, veterinary”[pt] OR “case reports”[pt] OR “clinical conference”[pt] OR “consensus development conference”[pt] OR “scientific integrity review”[pt])

### Study records

#### Data management

We will upload all papers retrieved from the databases to Rayyan [29]. This is an easy-to-use online platform where duplicate records from different bibliographic searches can be identified and subsequently removed. Decisions regarding the eligibility of studies can be recorded and compared across multiple reviewers in the space provided with the platform as well. Together, these features increase the quality of and reduce the amount of time needed to complete systematic reviews. By using Rayyan, we will also be able to maintain a searchable database of references related to the present systematic review, which will enable sharing information with other research groups and citation of important publications while writing up the future review paper.

#### Selection process

We will select eligible studies by screening titles and abstracts of all retrieved publications. Two pairs of independent reviewers will take part in the article screening phase, with each one skimming through half of all retrieved titles/abstracts. These researchers will be blinded to each other’s decisions and will resolve discrepancies through discussion or by consulting a fifth independent reviewer. In case titles and abstracts do not provide enough information to determine the eligibility of a particular study, the corresponding paper will be subjected to a full-text examination. Two reviewers will independently review all the full text, and they will be blinded to each other’s decisions. Any disagreements between them will be solved by a third reviewer. We will document reasons for any exclusion at this stage separately. Reviewer calibration will be done prior to title/abstract screening and the study selection process by reviewing a random sample of 5% of the total number of citations. We will determine screening reliability by estimating a kappa score among all reviewers.

#### Data collection process

One reviewer will extract data from the selected publications, and their work will be supervised by another independent member of the research team. A customized data extraction tool will be proposed, based on the included articles. Key variables and outcomes listed in the tool will be pilot tested on a random sample of eligible studies and this tool will be revised until consensus is reached. The supervisor will double-check data collected from the original studies in order to detect typing errors or problems of interpretation. We will extract data from the text and tables of the included articles, but in case this is not possible the study authors will be contacted by e-mail to request any missing or additional information. All extracted information will be recorded in a spreadsheet. Unless papers include unique combinations of interventions and oral health outcomes, the publications reporting on data from the same study will be included only once in the review synthesis. In this case, the most recent publication will be reviewed.

### Data items

We will collect data according to the five different blocks of information that follow:

1. Study identification: authors, title, year, language, country of publication, and contact author e-mail;
2. Study characteristics: study location and design, who conducted and who delivered the intervention, sampling technique/method, age, race, and gender of participants, data collection method, case definition, number of participants in the experimental group, presence and number of participants in the control group, control definition, power calculation reported, and randomization/blinding;
3. Intervention characteristics: level of intervention (i.e., intrapersonal, interpersonal, institutional/systemic/structural racism), timing of intervention, duration of intervention, and follow-up period;
4. Outcome measures: all clinically-determined and self-reported oral health outcomes will be analyzed, such as reduction or improvement in dental caries or periodontal diseases, self-rated oral health/oral health-related quality of life. Whenever possible, either discrete or continuous numerical data will be extracted for the outcomes, even though some might have been measured categorically. In this case, relative and absolute frequencies will be extracted; and
5. Other: drop-outs and overall results of the study.

### Outcomes and prioritization

We will extract data on any oral health outcome measure, be they clinically assessed or self-reported. No particular outcome will be given priority in the analysis and synthesis of results.

### Risk of bias in individual studies

Two pairs of independent reviewers will be available for article assessment by using collectively pre-defined criteria. Each pair will assess half of all included studies, and any discrepancies will be resolved through discussion or by consulting a fifth independent investigator. While we will assess randomized controlled trials (RCTs) with the “Risk-of-Bias 2” (RoB-2) tool [30], non-randomized trials will be assessed with the “Risk of Bias in Non-randomized Studies – of Interventions” (ROBINS-I) instrument [31]. As for observational studies, these will be assessed with the “Newcastle-Ottawa Scale” (NOS) according to study design [32]. RoB-2 evaluates features of the trial design, conduct, and reporting, including randomization process, outcome measurement, and adherence to some pre-specified analysis plan. Each outcome of the trial is assessed for the potential risk of bias through a series of questions pertaining to different domains. Based on the answers, an overall judgment of “low” or “high” risk of bias, or “some concerns of bias,” is generated by an algorithm [30]. ROBINS-I also evaluates a fixed set of biases and provides an overall assessment of the risk of bias. It covers pre-intervention domains, which include confounding and selection biases, at-intervention domain, focusing on information bias, and post-intervention domains, namely confounding, selection, information, and reporting bias. For each individual bias domain, a set of questions estimates the risk of bias, with the following possible judgments: “low,” “moderate,” “serious” or “critical” risk of bias, or “no information.” The overall risk of bias derives from a combination of the domains, and it is at least as severe as the most serious risk identified in a particular domain [31]. The NOS focuses on case-control and cohort studies by assessing selection and comparability characteristics in both epidemiologic designs. While the tool for cohort studies emphasizes outcome assessment, the instrument for case-control studies focuses on ascertainment of exposure. Each tool comprises 8 items, and studies can be awarded a maximum of one point for each item within the Selection and Exposure/Outcome categories, and a maximum of two points for Comparability [32].

### Data synthesis

Studies meeting the inclusion criteria will be qualitatively summarized first and then analyzed statistically. If possible, we will carry out a meta-analysis with subgroup and sensitivity analysis for all outcome measures with Stata, v.16.1 [33]. We will use a random effects model to determine the pooled standardized mean difference (for continuous outcomes) or risk ratio/absolute risk difference (for categorical outcomes) with 95% confidence intervals. No restrictions in terms of heterogeneity will be applied. Instead, we will investigate sources of heterogeneity through sensitivity and subgroup analysis on the basis of study design, geographical location, participant’s age, race, and gender, as well as methodological quality of the included studies. Standard chi-squared, Tau squared and *I*^2^ tests will be used for this purpose. The *I*^2^ index will be interpreted as low, moderate, or high inconsistency, if the values are equal to 25%, 50%, and 75%, respectively [34]. Formal meta-regression will also be conducted to address potential heterogeneity and inconsistency [35]. If the meta-analysis is not feasible, we will only provide a narrative synthesis of the review findings, including study characteristics, types of intervention, participant characteristics, and outcome characteristics.

### Meta-biases

In case we find at least 10 similar studies, visual inspection of funnel plots, along with Egger’s tests, will enable assessment of whether publication bias underlies the emerging patterns of findings.

### Confidence in cumulative evidence

If possible, the strength of the body of evidence for each outcome will be assessed with the Grading of Recommendations Assessment, Development, and Evaluation (GRADE) system [36]. In the GRADE assessment, the most important feature to be rated is the study design. In general, RCTs are treated as high-quality evidence, while observational studies are considered low-quality evidence. Subsequently, reviewers evaluate a set of potential limitations and strengths of the body of evidence, which can modify the initially rated quality of evidence. The five criteria, which can bring the quality of the evidence down, are the risk of bias of the original studies, inconsistency of results, indirectness of evidence, imprecision, and publication bias. Large magnitude of effect, examination of all plausible confounding factors, and dose-response gradients can increase the quality of the evidence, on the other hand. Following these previously established criteria, we will classify the quality of evidence for each oral health outcome as high, moderate, low, or very low [36]. As the GRADE approach is not a quantitative system for grading the quality of evidence, all decisions to downgrade or upgrade the body of evidence will be explicitly documented and made transparent to readers.

## Discussion

This systematic review is premised upon the concept that racism not only shapes population patterns of health, disease distribution, and related inequities, but also the questions we ask, the methods we use to answer them, and how we interpret research findings. Known as the fourth core construct of ecosocial theory [37], accountability and agency center around power differentials across multiple societal levels, and institutions’ and people’s capacity to act and be responsible for both actions taken and not taken. When it comes to framing and conducting epidemiologic research, this construct is useful to explain how racism makes scholars prioritize the measurement of racial health inequities and their psychosocial determinants over broader anti-racist interventions. In the field of dentistry, the dearth of studies on how to successfully mitigate racial oral health inequities is so extreme that none have been identified in previous literature reviews [8, 14, 15]. This raises serious doubts about whether the effectiveness of anti-racist interventions for reducing general health inequities extends to oral health inequities. It is precisely this knowledge gap that the present review aims to address. To this end, we will consider a broad range of oral health conditions, using a highly sensitive search strategy (see the “Methods” section above).

In particular, our systematic review has been established to answer the following three questions: Are there any interventions to mitigate racial oral health inequities or improve the oral health of racially marginalized groups? If so, how successful have they been at promoting racial oral health equity? How do conclusions of previous reviews change by taking the findings of oral health interventions into account? While a number of individual-level interventions focused on improving the dental status of racially marginalized groups might have been carried out, we expect that only a few have been devised to reduce racial oral health inequities per se. This would be consistent with the overall trend in the oral health literature referred to in the “Background” section, which emphasizes measuring racial gaps with methodological rigor over developing strategies to reduce the problem. Accordingly, successful initiatives to combat racial oral health inequities will likely be characterized by the short-term effects of either clinical (e.g., application of fluoride varnishes) or psychosocial (e.g., motivational interviewing) interventions, as they apply to members of a particular racialized group. We anticipate that broader interventions involving population aggregates will not be specifically targeted at racism, as it operates on institutional, systemic, and structural levels. Rather, these larger oral health interventions will more likely be based on, for instance, the equitable distribution of fluorides or the increased access to or use of oral health services, the impacts of which will presumably be negligible on racial oral health inequities. Taken together, reviewed studies will likely add to the conclusions of previous critical appraisals of the literature [8, 14, 15] by showing that while some specific dental interventions might momentarily diminish racial oral health inequities, these need to be complemented by other large-scale, society-level initiatives that tackle racism at other conceptual levels, different life domains, and its intersections with related systems of oppression. Our systematic review may indicate that a pressing knowledge gap to be filled relates to interventions aimed to reduce racial inequities in a handful of domains, such as the labor market, the criminal justice system, and the school system, for example [7]. As has long been proffered by public health [8] and social science [28, 38] scholars, complex problems—such as racial inequities in (oral) health—demand comprehensive solutions, particularly those designed by the communities themselves or in partnership with racially marginalized groups.

Our systematic review will thus help build an evidence base which may inform policies and actions against all forms of racial injustice in the health field. By bringing together general and oral health interventions, we hope to advance a stronger anti-racist narrative, without which researchers, policymakers, and other social actors cannot construct fairer and healthier societies. The findings and conclusions that will emerge from this review will be disseminated in peer-reviewed publications, scientific conferences, social media, and press releases. Doing so is only one small step towards improved racial (health) equity, however [39].

## Data Availability

Not applicable.
