# Correction: Where are race-based oral health inequities bound? Protocol for a systematic review on interventions to tackle racial injustice in dental outcomes

**DOI:** 10.1186/s13643-022-02009-z

**Published:** 2022-06-24

**Authors:** João L. Bastos, Helena M. Constante, Helena S. Schuch, Dandara G. Haag, Sonia Nath, Roger K. Celeste, Carol C. Guarnizo-Herreño, Mary J. McCallum, Lisa M. Jamieson

**Affiliations:** 1 Federal University of Santa Catarina, Florianópolis, Brazil; 2 Federal University of Pelotas, Pelotas, Brazil; 3 Australian Research Centre for Population Oral Health, The University of Adelaide, Adelaide, Australia; 4 Department of Preventive and Social Dentistry, Federal University of Rio Grande do Sul, Porto Alegre, Brazil; 5 Facultad de Odontología, Universidad Nacional de Colombia, Bogotá, Colombia; 6 Dentist of Cree Descent, Winnipeg, Canada

**Correction: Syst Rev 11, 41 (2022)**

10.1186/s13643-022-01911-w

Following publication of the original article [1], the authors identified an error in the author name of Carol C. Guarnizo-Herreño.

The incorrect author name is: Carol C. Guanizo-Herreño

The correct author name is: Carol C Guarnizo-Herreño

The author group has been updated above and the original article [1] has been corrected.
